# Serum Apelin‐13 and Galectin‐3 in COVID‐19: Associations With Routine Biochemical Parameters and Diagnostic Performance in a Case–Control Study

**DOI:** 10.1002/jcla.70284

**Published:** 2026-06-19

**Authors:** İsmail Uğurlu

**Affiliations:** ^1^ Department of Medical Biochemistry, Faculty of Medicine Kafkas University Kars Türkiye

**Keywords:** Apelin‐13, COVID‐19, Galectin‐3, routine laboratory testing

## Abstract

**Background:**

Apelin‐13 and Galectin‐3 (Gal‐3) are bioactive mediators linked to inflammation and cardiometabolic pathways. Their clinical utility in COVID‐19 alongside routine laboratory tests remains unclear.

**Methods:**

In this case–control study, 89 adults were enrolled (COVID‐19 negative controls, *n* = 44; COVID‐19 positive patients confirmed by PCR and/or CT, *n* = 45). Serum Apelin‐13 and Gal‐3 were measured by ELISA, and routine biochemical parameters were obtained using automated analyzers. Group comparisons and correlation analyses were performed, and receiver operating characteristic (ROC) analysis assessed discriminatory performance.

**Results:**

Compared with controls, the COVID‐19 group showed higher ALT (*p* = 0.046), GGT (*p* = 0.004), ALP (*p* = 0.001), total bilirubin (*p* = 0.008), direct bilirubin (*p* = 0.001), urea (*p* = 0.001), creatinine (*p* = 0.001), glucose (*p* = 0.001), and CRP (*p* = 0.001), and lower eGFR (*p* = 0.001), albumin (*p* = 0.010), and total cholesterol (*p* = 0.021). In controls, Apelin‐13 correlated negatively with urea (*r* = −0.333, *p* = 0.027) and Gal‐3 correlated negatively with cholesterol (*r* = −0.342, *p* = 0.023). In COVID‐19 patients, Apelin‐13 correlated positively with glucose (*r* = 0.320, *p* = 0.032) and negatively with sodium (*r* = −0.323, *p* = 0.030). ROC analysis showed limited diagnostic value for Gal‐3 (AUC = 0.549; *p* = 0.426) and Apelin‐13 (AUC = 0.533; *p* = 0.594).

**Conclusions:**

Apelin‐13 and Gal‐3 showed limited associations with selected biochemical alterations in COVID‐19, but neither biomarker demonstrated adequate standalone discriminatory performance.

## Graphical abstract

Serum Apelin‐13 and Galectin‐3 levels were measured by ELISA in COVID‐19–positive patients and controls and evaluated alongside routine biochemical parameters. COVID‐19 patients showed significant alterations in liver, renal, inflammatory, and metabolic markers. Apelin‐13 and Galectin‐3 demonstrated limited correlations with selected biochemical variables but showed no significant diagnostic performance in ROC analysis.

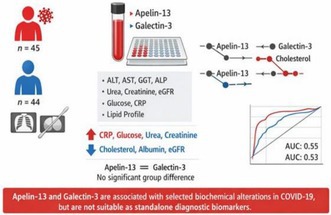

## 1 Introduction

COVID‐19 disease as a cluster of pneumonia cases of unknown etiology in Wuhan, China, towards the end of 2019 [1]. One month after its initial diagnosis, it spread nationwide [2], quickly spread globally [1], placing heavy burdens on both healthcare systems and social order [3]. In the early stages of the outbreak, societies were caught unprepared because immunity to this virus was virtually nonexistent, resulting in high rates of transmission, severe complications, and mortality. Mortality rates have reached very high levels in some societies, with factors such as age and the presence of chronic diseases contributing to the impact [4]. The challenges and uncertainties brought about by the pandemic have necessitated in‐depth research into the pathophysiology of COVID‐19 and the effects of the infection on various parameters [5].

The discovery of the coronavirus family dates back to the B814 virus, isolated from children with upper respiratory tract infections in the 1960s [6, 7, 8]. Subsequently, research continued with the identification of similar viruses such as 229E, NL63, HKU1, and OC43 in common colds by various researchers [9, 10, 11]. Over time, coronaviruses have become versatile pathogens, capable of causing a wide range of diseases, from mild upper respiratory tract illnesses to severe acute respiratory syndromes [12]. They are reported to have significant morbidity and mortality rates [13]. When examining the infection mechanism and evolutionary process, the transmission from animal populations to humans and their mutational abilities are factors that maintain the epidemic potential of these viruses [14, 15]. Understanding the pathophysiological effects of this virus family is important for clinical management. Indeed, it has been reported that approximately 80% of COVID‐19 patients present with mild or moderate symptoms, while the remaining 20% can develop severe clinical outcomes such as severe pneumonia, hypoxia, septic shock, or multiorgan failure [16, 17, 18, 19]. Processes such as pulmonary edema, endothelial damage, and widespread inflammatory response are among the multisystem effects of COVID‐19 [20, 21].

Apelin‐13 is defined as a peptide adipokine discovered as the endogenous ligand of the apelin receptor [APJ] and associated with cardiovascular functions and fluid‐electrolyte balance [22]. Galectin‐3, on the other hand, is a versatile protein that can bind to β‐galactoside cell [23]. Increased cytokine and chemokine production during infection supports the excessive inflammatory response referred to as the “cytokine storm.” During this process, endothelial cells, monocytes, and macrophages are activated, and it has been reported that numerous biological mediators are also activated [24].

The role of molecules such as Apelin‐13 and Galectin‐3, which are involved in inflammatory processes, in the pathophysiology of the disease is being investigated. Altered serum levels of both biomolecules are thought to provide clues about the body's response to infection. Apelin‐13 has been reported to have various physiological roles, ranging from the cardiovascular system to metabolic processes [25], and Galectin‐3 has been reported to have effects on many pathological processes, including inflammation and fibrosis [23, 26]. Therefore, in a systemic disease such as COVID‐19, collecting and evaluating data on these two biomarkers is important for both clinical practice and research and development [27]. Although the apelin family plays a systemic role in vascular vasodilation, blood pressure regulation, and metabolic homeostasis, the galectin family has been reported to have the capacity to modulate the inflammatory response and fibrotic processes [25, 27].

This study aimed to investigate the relationships between serum levels of Apelin‐13 and Galectin‐3 and some other routine biochemical parameters in individuals diagnosed with COVID‐19. The biochemical processes of COVID‐19 are thought to manifest themselves through potential regulatory mechanisms, and this study sought to identify new markers that could contribute to the pathophysiology of the disease.

## 2 Methods

The study was conducted with 45 patients (M: 28; F: 17) over the age of 18, without any chronic disease, and with a definitive COVID‐19 diagnosis by PCR (+) and/or CT (+) who presented to the Kars Harakani State Hospital with suspected COVID‐19. The control group consisted of 44 individuals (M: 22; F: 22) over the age of 18, who presented to the hospital for checkups without any chronic disease and had not had COVID‐19. A total of 89 volunteers participated. All sociodemographic and clinical data of the participating individuals were recorded at the bedside. Routine biochemistry parameters were retrieved from the hospital database in accordance with the permissions obtained from the relevant centers and analyzed.

Participant blood samples were centrifuged at 4000 rpm for 15 min at +4°C. Biochemical parameter measurements included liver function tests including AST, ALT, GGT, LDH, ALP activities, total bilirubin, and direct bilirubin levels; kidney function tests including eGFR, urea, creatinine, albumin, total protein, and uric acid levels; HDL, LDL, cholesterol, and triglyceride levels; fluid electrolyte parameters including sodium, potassium, calcium, magnesium, phosphorus, and chloride; and other routine parameters including glucose and CRP levels, as well as amylase and creatine kinase activities, which were performed by autoanalyzer. After routine biochemical parameter analyses were performed on the serum obtained, the remaining portion was aliquoted into Eppendorf tubes and the required samples were stored at −80°C until the day of the study for Enzyme‐Linked Immunosorbent Assay (ELISA) studies.

**FIGURE 1:**
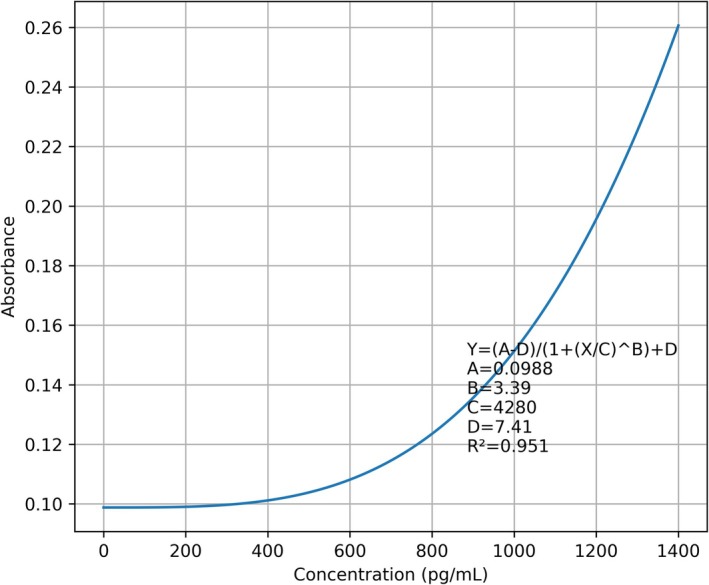
Serum Apelin‐13 standard curve.

**FIGURE 2:**
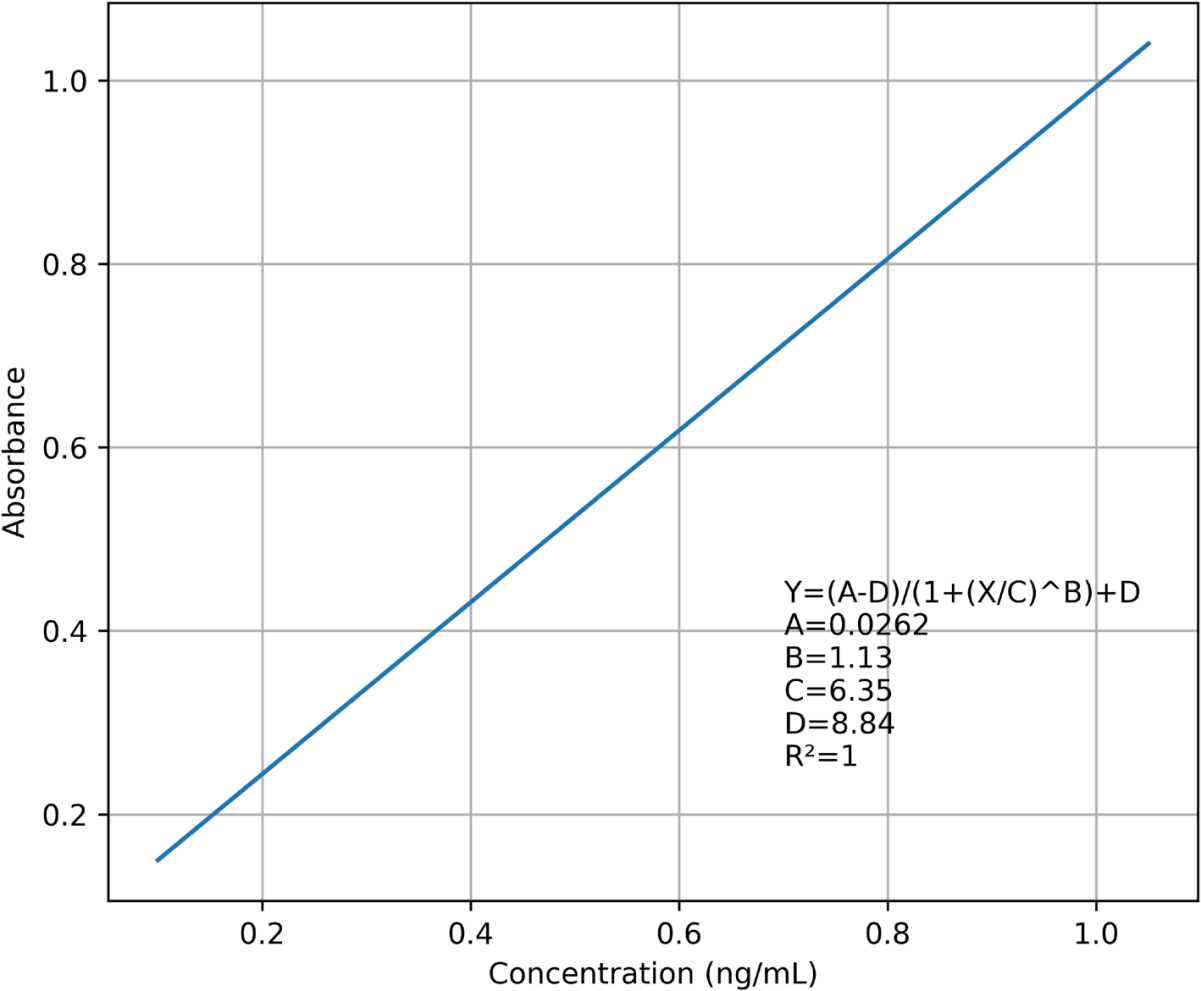
Serum Gal‐3 standard curve.

Serum Apelin‐13 (Human apelin 13, AP13 ELISA Kit, 96 Tests, SunLong, Catalog No.: SL0279Hu, Lot No.: 20220816, Hangzhou, China) and Gal‐3 (Human Galectin‐3, GAL‐3 ELISA Kit, 96 Tests, AFG Bioscience, Catalog No.: EK711634, Lot No.: O4282, USA) concentrations of relevant parameters were measured using commercial ELISA kits based on the non‐competitive sandwich ELISA method. Reagents, standard solutions, and samples in the kits were prepared according to the manufacturers' instructions and a total of 89 serum samples were analyzed. Samples were incubated at 37°C, then washed with an ELISA plate washer (Bio‐tek ELX50, USA), and after the addition of Streptavidin‐HRP, the color reaction was measured at 450 nm with an ELISA plate reader (Epoch, BioTek, USA). Apelin‐13 levels were evaluated in the range of 30–1500 pg/mL (sensitivity 8 pg/mL; Figure 1), and Gal‐3 levels were evaluated in the range of 0.7–22 ng/mL (sensitivity 0.1 ng/mL; Figure 2).

Statistical analyses were performed using IBM SPSS Statistics version 26.0 (IBM Corp., Armonk, NY, USA). The normality of continuous variables was assessed using the Shapiro–Wilk test.

Normally distributed variables were expressed as mean ± standard deviation (SD), whereas non‐normally distributed variables were presented as median (minimum–maximum). Comparisons between the COVID‐19 patient group and the control group were performed using the Independent Samples *t*‐test for normally distributed variables and the Mann–Whitney *U* test for non‐normally distributed variables.

Correlation analyses were conducted to evaluate the relationships between serum Apelin‐13 and Galectin‐3 levels and routine biochemical parameters, including liver function tests (AST, ALT, GGT, ALP, total bilirubin, direct bilirubin), renal function parameters (urea, creatinine, eGFR, albumin, uric acid), lipid profile parameters (total cholesterol, triglycerides, HDL cholesterol, LDL cholesterol), electrolyte parameters (sodium, potassium, chloride, calcium, phosphorus, magnesium), and other laboratory parameters (glucose, CRP, amylase, and creatine kinase).

Pearson correlation analysis was used for variables with normal distribution. Since non‐parametric correlation analysis was not ultimately applied in the final statistical evaluation, Spearman correlation analysis was removed from the statistical methods section to avoid inconsistency with the presented tables.

Receiver operating characteristic (ROC) curve analysis was performed to evaluate the diagnostic performance of Apelin‐13 and Galectin‐3 in distinguishing COVID‐19 patients from healthy controls. The area under the curve (AUC), sensitivity, specificity, and 95% confidence intervals were calculated.

A *p*‐value < 0.05 was considered statistically significant.

## 3 Results

A total of 89 participants were included in the study. Of these participants, 45 (28 males, 17 females) were assigned to the patient group and 44 (22 males, 22 females) to the control group (Table 1). No statistically significant difference was found between the groups in terms of gender (*p* = 0.357).

**TABLE 1:** Comparison of demographic data of participants.

Variable	Control	Patient	Total	*p*
Gender (*n*, %)	Female: 22 (50)	Female: 17 (37.70)	39	0.357*
	Male: 22 (50)	Male: 28 (62.30)	50	

* Chi‐square test (*p* < 0.05).

The results regarding the biochemical parameters of the participants are presented in Table 2.

**TABLE 2:** Evaluation of the biochemical parameters of the participants.

Parameter	Control (*n* = 44) [mean ± SD (median)]	Patient (*n* = 45) [mean ± SD (median)]	*p*
AST (U/L)	21.39 ± 10.14 (17)	26.15 ± 15.55 (25)	0.200
ALT (U/L)	18.82 ± 12.54 (13.5)	28.23 ± 23.04 (19)	0.046*
LDH (U/L)	196.73 ± 24.72 (202)	198.21 ± 21.10 (201)	0.964
GGT (U/L)	30.23 ± 18.01 (28)	41.12 ± 12.09 (43)	0.004*
ALP (U/L)	73.20 ± 21.47 (71)	91.26 ± 26.90 (85)	0.001*
Total bilirubin (mg/dL)	0.54 ± 0.40 (0.39)	0.73 ± 0.42 (0.60)	0.008*
Direct bilirubin (mg/dL)	0.19 ± 0.13 (0.16)	0.31 ± 0.22 (0.23)	0.001*
Urea (mg/dL)	28.78 ± 10.31 (26)	49.21 ± 21.53 (50)	0.001*
Creatinine (mg/dL)	0.77 ± 0.19 (0.76)	1.00 ± 0.48 (1.07)	0.001*
eGFR (mL/min/1.73 m^2^)	99.99 ± 21.65 (101)	57.30 ± 30.90 (62.02)	0.001*
Uric acid (mg/dL)	5.28 ± 1.13 (5.0)	5.10 ± 1.39 (5.4)	0.161
Albumin (g/dL)	40.56 ± 7.00 (42.2)	37.50 ± 5.17 (36.0)	0.010*
Total protein (g/dL)	65.19 ± 5.67 (64.5)	64.60 ± 6.22 (64.0)	0.335
Cholesterol (mg/dL)	179.93 ± 29.60 (174)	166.30 ± 30.57 (160)	0.021*
Triglyceride (mg/dL)	180.80 ± 73.78 (208)	160.77 ± 77.18 (127)	0.121
HDL (mg/dL)	45.43 ± 13.25 (42.5)	45.11 ± 11.52 (40)	0.869
LDL (mg/dL)	102.48 ± 27.69 (93)	105.30 ± 30.58 (90.6)	0.586
Sodium (mmol/L)	138.07 ± 3.49 (139)	137.40 ± 4.35 (138)	0.417
Potassium (mmol/L)	4.45 ± 0.45 (4.52)	4.00 ± 0.68 (4.5)	0.493
Chloride (mmol/L)	100.96 ± 4.05 (102)	100.40 ± 4.24 (100.9)	0.397
Calcium (mg/dL)	8.59 ± 0.78 (8.55)	8.00 ± 0.66 (8.2)	0.225
Phosphorus (mg/dL)	3.53 ± 0.37 (3.43)	3.00 ± 0.52 (3.6)	0.261
Magnesium (mg/dL)	1.98 ± 0.13 (1.94)	2.00 ± 0.15 (2.03)	0.274
Glucose (mg/dL)	98.02 ± 13.11 (97.5)	137.64 ± 64.75 (114)	0.001*
Amylase (U/L)	53.18 ± 18.23 (52)	58.17 ± 17.98 (52)	0.284
Creatine kinase (U/L)	95.80 ± 88.35 (73)	119.11 ± 106.42 (90)	0.257
CRP (mg/L)	27.03 ± 37.37 (17.13)	80.80 ± 80.84 (53.53)	0.001*

Abbreviations: M, median; Mean, mean value; SD, standard deviation.

* Mann–Whitney *U* test; *p* < 0.05 considered statistically significant.

Table 2 shows that liver parameters such as ALT, GGT, ALP, and total bilirubin and direct bilirubin were significantly higher in the patient group compared to the control group (*p* < 0.05). When the patient and control groups were compared regarding kidney parameters, a statistically significant difference was found in terms of urea, creatinine, eGFR, and albumin levels (*p* < 0.05). When the lipid profile parameters were examined, a significant difference was found between the groups only in terms of cholesterol levels. Cholesterol levels in the patient group were found to be statistically significantly lower compared to the control group (*p* = 0.021). No significant difference was observed between the two groups in terms of triglyceride, HDL Cholesterol, and LDL Cholesterol levels (*p* < 0.05). In this study, no statistically significant difference was observed between the patient and control groups in terms of electrolyte values (sodium, potassium, chloride, calcium, phosphorus, magnesium) (*p* < 0.05). In the study, glucose and CRP values were found to be statistically significantly higher in the patient group compared to the control group (*p* < 0.05). The correlation analysis results between Gal‐3 and Apelin‐13 and Biochemical Parameters of the control group are presented in Table 3.

**TABLE 3:** Correlation of Galectin‐3 and Apelin‐13 with laboratory parameters in the control group.

Parameter	Gal‐3 (ng/mL)		Apelin‐13 (pg/mL)	
	*r*	*p*	*r*	*p*
AST (U/L)	−0.091	0.556	−0.209	0.173
ALT (U/L)	0.071	0.646	−0.083	0.591
LDH (U/L)	0.292	0.055	0.085	0.582
GGT (U/L)	0.094	0.546	−0.028	0.855
ALP (U/L)	−0.008	0.960	0.210	0.171
Total bilirubin (mg/dL)	0.161	0.297	0.020	0.898
Direct bilirubin (mg/dL)	0.230	0.134	0.044	0.778
Urea (mg/dL)	−0.138	0.372	−0.333	0.027*
Creatinine (mg/dL)	0.094	0.543	0.068	0.661
eGFR (mL/min/1.73 m^2^)	−0.135	0.382	−0.135	0.381
Uric acid (mg/dL)	0.103	0.506	−0.200	0.194
Albumin (g/dL)	−0.006	0.967	0.082	0.595
Total protein (g/dL)	−0.069	0.658	0.030	0.848
Cholesterol (mg/dL)	−0.342	0.023*	−0.015	0.924
Triglyceride (mg/dL)	−0.294	0.053	0.003	0.987
HDL (mg/dL)	−0.033	0.832	−0.050	0.746
LDL (mg/dL)	0.281	0.065	−0.038	0.804
Sodium (mmol/L)	−0.123	0.425	−0.136	0.380
Potassium (mmol/L)	0.005	0.975	−0.115	0.457
Chloride (mmol/L)	0.026	0.869	−0.185	0.230
Calcium (mg/dL)	0.011	0.942	−0.099	0.522
Phosphorus (mg/dL)	−0.160	0.299	0.014	0.926
Magnesium (mg/dL)	−0.085	0.584	−0.142	0.356
Glucose (mg/dL)	−0.238	0.121	0.040	0.794
Amylase (U/L)	0.013	0.931	−0.013	0.931
Creatine kinase (U/L)	0.075	0.629	0.070	0.650
CRP (mg/L)	0.009	0.955	−0.113	0.464

* Pearson correlation analysis (*p* < 0.05); *r*: Pearson correlation coefficient.

In Table 3, no statistically significant correlation was found between Gal‐3 and Apelin‐13 and the control group liver laboratory parameters (AST, ALT, LDH, GGT, ALP, total bilirubin, direct bilirubin) (*p* > 0.05). No statistically significant correlation was found between Gal‐3 and routine lipid profile parameters such as triglycerides, HDL and LDL (*p* > 0.05). Only a low to moderate negative correlation was found between cholesterol and Gal3 (*r* = −0.342, *p* = 0.023). At the same time, no statistically significant correlation was found between routine lipid profile parameters and Apelin‐13 (*p* > 0.05). No statistically significant correlation was found between Gal‐3 and the renal parameters urea, creatine, eGFR, uric acid, and albumin in the control group (*p* > 0.05). A low to moderate negative correlation was found between Apelin‐13 and urea levels (*r* = −0.333, *p* = 0.027). No statistically significant correlation was found between other renal parameters (creatinine, eGFR, uric acid, albumin) and Apelin‐13 in the control group (*p* > 0.05). Neither Gal‐3 nor Apelin‐13 showed statistically significant correlation with the control group fluid electrolyte parameters sodium, potassium, chloride, calcium, phosphorus, magnesium, nor with other routine laboratory parameters such as glucose, amylase, creatine kinase, and CRP (*p* > 0.05). The correlation analysis results between Gal‐3 and Apelin‐13 and biochemical parameters of the patients group are presented in Table 4.

**TABLE 4:** Correlation of Galectin‐3 and Apelin‐13 with laboratory parameters in the patient group.

Parameter	Gal‐3 (ng/mL)		Apelin‐13 (pg/mL)	
	*r*	*p*	*r*	*p*
AST (U/L)	−0.099	0.519	0.085	0.581
ALT (U/L)	0.030	0.844	−0.024	0.875
LDH (U/L)	−0.054	0.725	−0.139	0.363
GGT (U/L)	0.060	0.696	0.157	0.302
ALP (U/L)	−0.051	0.738	−0.020	0.894
Total bilirubin (mg/dL)	−0.160	0.294	0.143	0.350
Direct bilirubin (mg/dL)	0.254	0.092	0.161	0.290
Urea (mg/dL)	0.258	0.087	−0.258	0.087
Creatinine (mg/dL)	0.018	0.908	0.176	0.248
eGFR (mL/min/1.73 m^2^)	−0.007	0.962	−0.137	0.369
Uric acid (mg/dL)	0.077	0.615	0.268	0.075
Albumin (g/dL)	−0.152	0.319	0.290	0.054
Total protein (g/dL)	−0.103	0.500	0.270	0.073
Cholesterol (mg/dL)	0.040	0.793	−0.096	0.530
Triglyceride (mg/dL)	0.059	0.699	0.025	0.869
HDL (mg/dL)	−0.205	0.178	−0.112	0.465
LDL (mg/dL)	0.241	0.110	−0.070	0.649
Sodium (mmol/L)	0.005	0.975	−0.323	0.030*
Potassium (mmol/L)	0.137	0.369	0.083	0.586
Chloride (mmol/L)	−0.150	0.326	−0.278	0.065
Calcium (mg/dL)	0.018	0.904	0.008	0.959
Phosphorus (mg/dL)	−0.113	0.458	0.174	0.253
Magnesium (mg/dL)	−0.213	0.160	0.130	0.395
Glucose (mg/dL)	−0.086	0.574	0.320	0.032*
Amylase (U/L)	−0.259	0.085	−0.254	0.092
Creatine kinase (U/L)	−0.159	0.298	−0.161	0.292
CRP (mg/L)	−0.203	0.181	0.193	0.205

* Pearson correlation analysis (*p* < 0.05); *r*: Pearson correlation coefficient.

**FIGURE 3:**
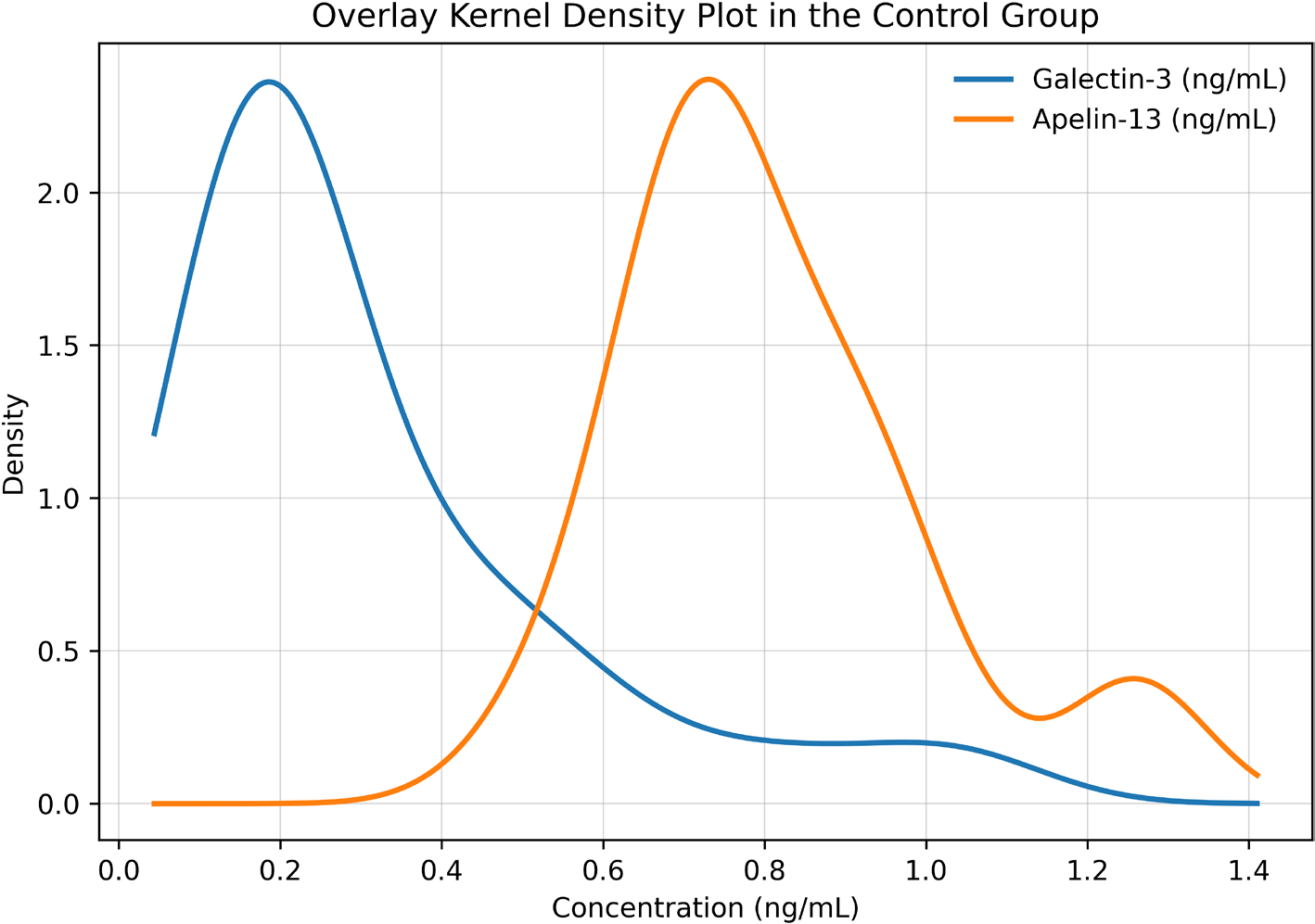
Overlay kernel density plot in the control group (Apelin‐13, pg/mL values converted to ng/mL).

Table 4 shows that in the COVID‐19 patient group, there was no statistically significant correlation between Gal‐3 and Apelin‐13 levels with AST, ALT, LDH, GGT, ALP, total bilirubin, and direct bilirubin values (*p* > 0.05). No statistically significant correlation was found between Gal‐3 and Apelin‐13 among the routine lipid profile parameters such as cholesterol, triglycerides, HDL, and LDL (*p* > 0.05) (Table 4). In the patient group, a statistically significant, low‐moderate negative correlation was found between Apelin‐13 and sodium levels (*r* = −0.323, *p* = 0.030) (Table 4). No significant correlation was found between Gal‐3 and Apelin‐13 and fluid electrolyte parameters (potassium, chloride, calcium, phosphorus, magnesium) (*p* > 0.05) (Table 4). In the patient group, there was no significant correlation between Gal‐3 and Apelin‐13 and other routine laboratory parameters such as amylase, creatine kinase, and CRP (*p* > 0.05) (Table 4). On the other hand, a statistically significant, low‐to‐moderate positive correlation was found between Apelin‐13 and glucose levels in the patient group (*r* = 0.320, *p* = 0.032). The overlay kernel density plot showing the distribution of serum Galectin‐3 and Apelin‐13 levels in the control group is presented in Figure 3.

**FIGURE 4:**
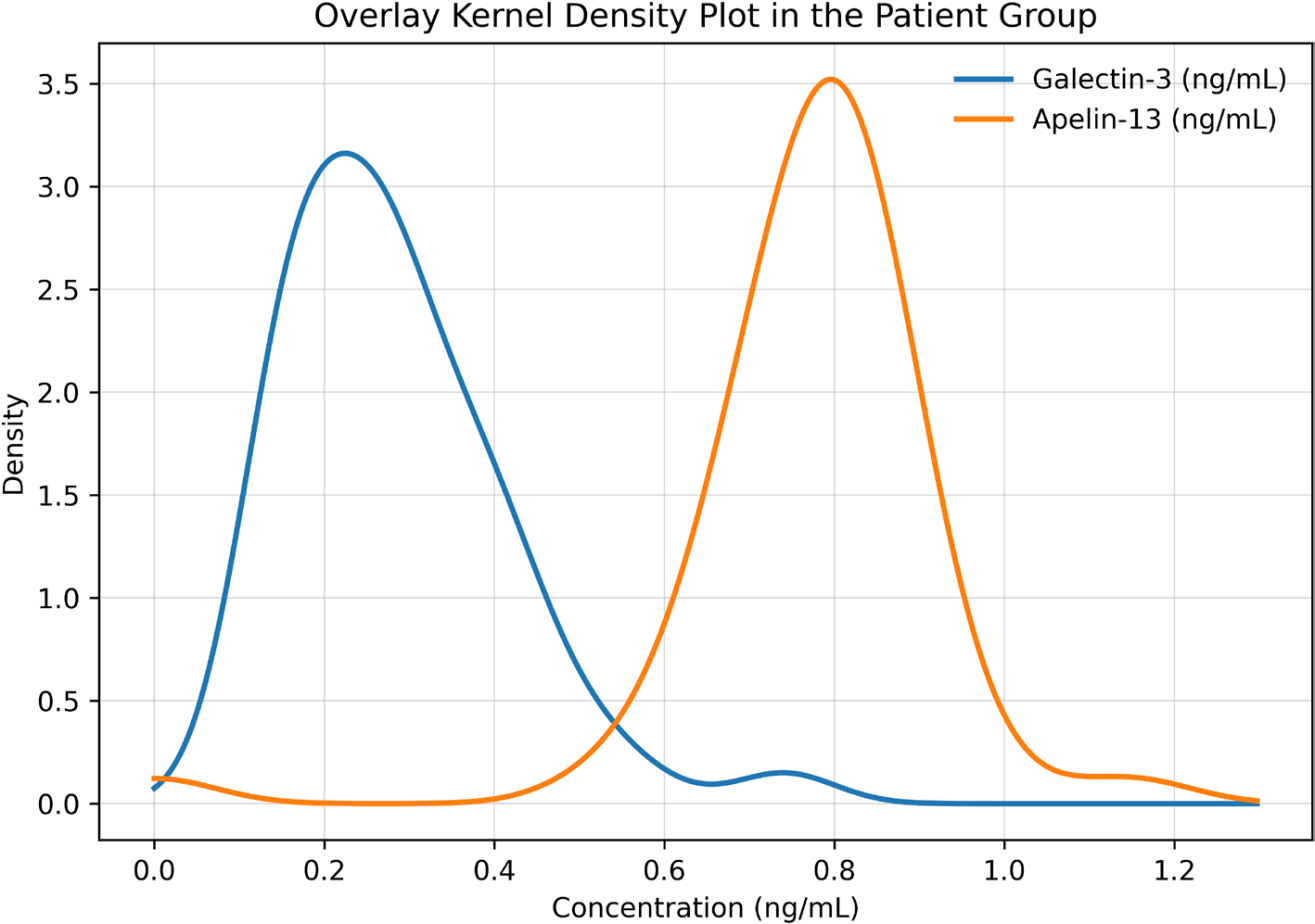
Overlay kernel density plot in the patient group (Apelin‐13, pg/mL values converted to ng/mL).

Figure 3 shows the concentration distributions of the two molecules in the control group as distinctly different. Galectin‐3 is concentrated at lower concentrations and in a narrower range, while Apelin‐13 has higher average concentrations and a broader, potentially bimodal, distribution. The lack of overlap between the curves confirms that the two biomarkers maintain distinct physiological ranges in healthy individuals; both have low variance, consistent with homeostatic stability. The overlay kernel density plot showing the distribution of serum Galectin‐3 and Apelin‐13 levels in the patient group is presented in Figure 4.

According to Figure 4, the concentration distributions of Galectin‐3 and Apelin‐13 differed in the patient group. Although Galectin‐3 remained at lower concentrations, Apelin‐13 had a higher average concentration and was concentrated around a narrower peak compared to the control group. Compared to the control group graph (Figure 3), the peaks of both molecules were slightly shifted to the right (toward higher concentrations) in the patient group. A comparative table of serum Gal‐3 and Apelin‐13 levels is presented in Table 5.

**TABLE 5:** Serum Galectin‐3 and Apelin‐13 levels.

Parameter	Control (*n* = 44) [mean ± SD (median)]	Patient (*n* = 45) [mean ± SD (median)]
Gal‐3 (ng/mL)	0.31 ± 0.24 (0.21)	0.30 ± 0.13 (0.26)
Apelin‐13 (pg/mL)	805.42 ± 187.80 (747.92)	792.10 ± 104.05 (791.69)

*Note:* Mann–Whitney *U* test; *p* < 0.05.

Abbreviations: M, median; Mean, mean; SD, standard deviation.

According to Table 5, no statistically significant difference was found between the patient and control groups in terms of Gal‐3 and Apelin‐13 parameters (*p* > 0.05). The correlation analysis results between Gal‐3 and Apelin‐13 levels in the control group are presented in Table 6.

**TABLE 6:** Correlation between Galectin‐3 and Apelin‐13 in the control group.

Parameter	Galectin‐3	
	*r*	*p*
Apelin‐13 (pg/mL)	0.481	0.001*

* Pearson correlation analysis (*p* < 0.05); *r*: Pearson correlation coefficient.

According to Table 6, a statistically significant moderate positive correlation was found between Gal‐3 and Apelin‐13 levels in the control group (*r* = 0.481, *p* = 0.001). The correlation analysis results between Gal‐3 and Apelin‐13 levels in the patient group are presented in Table 7.

**TABLE 7:** Correlation between Galectin‐3 and Apelin‐13 in the patient group.

Parameter	Galectin‐3	
	*r*	*p*
Apelin‐13 (pg/mL)	0.196	0.198

According to Table 7, no statistically significant correlation was found between Gal‐3 and Apelin‐13 in the patient group (*p* = 0.198).

**FIGURE 5:**
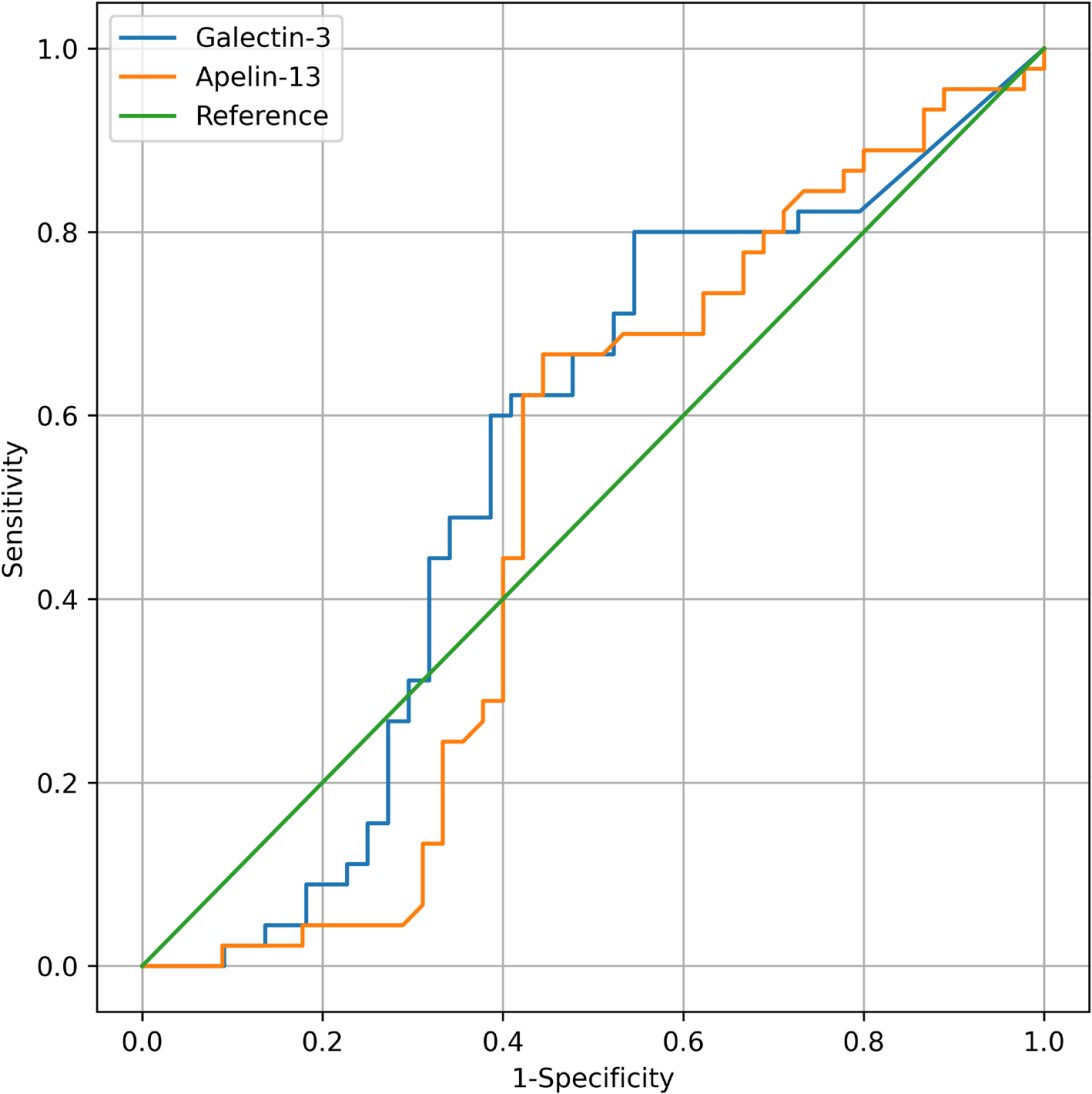
ROC analysis curves.

ROC analysis curves evaluating the ability of Galectin‐3 and Apelin‐13 biomarkers to distinguish disease are presented in Figure 5 and the results are presented in Table 8.

**TABLE 8:** ROC analysis of Galectin‐3 and Apelin‐13 for disease discrimination.

Biomarker	AUC	Cut‐off	Sensitivity	Specificity	95% CI	*p*
Galectin‐3	0.549	0.24	49%	41%	0.425–0.673	0.426
Apelin‐13	0.533	777	41%	43%	0.407–0.659	0.594

Abbreviations: AUC, area under the curve; CI, confidence interval.

According to Figure 5, the area under the curve (AUC) for Galectin‐3 was calculated as 0.549, indicating that the biomarker has a low ability to distinguish disease.

According to Table 8, the cut‐off value for Galectin‐3 was 0.24, with a sensitivity of 49% and a specificity of 41%. The 95% confidence interval was between 0.425 and 0.673, with a *p*‐value of 0.426. This *p*‐value indicates that there was no statistically significant difference. Similarly, the AUC value for the Apelin‐13 biomarker was calculated as 0.533, demonstrating a low ability to distinguish disease (Figure 5). The cut‐off value for Apelin‐13 was determined as 777, with a sensitivity of 41% and a specificity of 43%. The 95% confidence interval for this biomarker was between 0.407 and 0.659, with a *p* value of 0.594. This indicates that there was no statistically significant difference (Table 8).

## 4 Discussion

The COVID‐19 disease, which has affected millions of people worldwide and claimed millions of lives, spread from one city in China to the entire country in 30 days [28], causing pathophysiological conditions in the organs and systems of the human body and turning into an epidemic, posing significant clinical risks to the elderly and/or individuals with comorbidities [29]. By 2025, more than 777 million laboratory‐confirmed COVID‐19 patients have been identified, and more than 7 million deaths have been recorded by the WHO [30]. Since the COVID‐19 pandemic has become the global health crisis of the 21st century [31], reliable and easily accessible biomarkers are needed for rapid diagnosis, accurate treatment, and reducing the severity of the disease. The search for additional biochemical markers to facilitate both hospitalization decisions and the rapid identification of critically ill patients and referral to appropriate treatment has become a hot topic in the scientific world [5, 32]. The study was designed to reveal the possible connections between Apelin‐13 and Gal‐3 levels in COVID‐19 patients by examining them together with various routine biochemical parameters. Indeed, different data have been presented in the relevant literature regarding whether these two molecules provide a signal related to disease severity or prognosis [5, 27, 33].

In this context, 89 participants were examined [44 COVID‐19 negative and 45 COVID‐19 positive]. The mean age of the patient group was 68.58 ± 10.3 years, while the control group recorded it as 42.82 ± 14.6 years. This difference is consistent with the higher prevalence of COVID‐19, particularly in the elderly population, and with epidemiological data [4, 34, 35]. Literature data suggest that COVID‐19 can present with more severe symptoms in older populations and that management becomes more difficult with the presence of comorbid conditions [cardiovascular disease, hypertension, diabetes, COPD, lung cancer] [4, 28, 36]. A meta‐analysis conducted by Shi et al. [36], covering 27 observational studies, found that advancing age [60–70 years and older] significantly increased the risk of death. Wu and McGoogan, analyzing data from the Chinese Center for Disease Control and Prevention, reported that while the overall case fatality rate was 2%–3%, this rate rose to 8% in individuals aged 70–79 and 14.8% in individuals aged 80 and older [28]. The increasing prevalence of comorbidities with age in older COVID‐19 patients appears to be linked to increased disease severity and weakening of host defense mechanisms over time [29, 37]. Considering gender distribution, despite some publications suggesting that male gender poses a risk [37, 38], Gupta et al. [39] reported no association between disease severity and gender. Consistent with Gupta et al. [39], this study also found no significant difference between gender and disease, indicating that gender alone was not sufficient to determine disease course.

Liver enzyme activities and bilirubin levels have been reported as important indicators for understanding the potential effects of COVID‐19 on the liver [40, 41]. In this study, significant increases in ALT, GGT, and ALP activities suggest that SARS‐CoV‐2 infection may cause hepatocellular stress [36]. Mao et al. [42], approximately 20% of hospitalized COVID‐19 patients showed altered liver enzymes. Despite frequent elevations in ALT, AST, and GGT, significant ALP elevation was less common [42, 43]. In a pediatric cohort, at least one of ALT, AST, or GGT was elevated [44]. In contrast, differences in LDH and AST were not statistically significant in the present study. Shang et al. [45] reported that LDH elevation is associated. According to a Cochrane review evaluating routine laboratory tests in COVID‐19, LDH was significantly elevated in only a subset of studies [46]. In another report, LDH elevation was also observed in specific severe subgroups of patients [47]. Bertolini et al. [48] result of abnormal liver function tests at admission were common but did not frequently progress to severe liver failure. Bilirubin levels in liver function tests are indicators of hepatic clearance and biliary excretory capacity [49]. In this study, total and direct bilirubin levels were significantly increased. Serra et al. [50] reported hyperbilirubinemia in COVID‐19, while Feng et al. [44] observed increased direct bilirubin without significant change in total bilirubin. Bertolini et al. [48] also emphasized that abnormal liver tests in COVID‐19 are likely multifactorial. Galectin‐3 is known to participate in apoptosis, angiogenesis, fibrosis, and inflammation [5, 26, 27]. In this study, no significant correlation was found between Gal‐3, Apelin‐13, and liver parameters; however, further studies with larger cohorts and serial measurements have been recommended [41, 51].

Lipids such as cholesterol and triglycerides, whether dietary or liver‐synthesized, are transported via LDL and HDL. Systemic infections including COVID‐19 can significantly affect lipid profiles [52]. Cholesterol levels were significantly lower in the patients. Systematic reviews have highlighted associations between dyslipidemia and poor COVID‐19 prognosis [52, 53, 54]. Infection‐related cytokines [TNF, IL‐1, IL‐6] can alter lipid metabolism by upregulating LDL receptors in hepatocytes and changing lipoprotein turnover [55]. Hypolipidemia in COVID‐19 has also been attributed to inflammation‐driven vascular permeability and leakage of lipids into tissues [56]. Decreases in total cholesterol have been linked with disease severity and proposed as markers of infection‐related metabolic alterations [56]. Chidambaram et al. [57] reported that LDL and HDL modulate viral interactions with host cell membranes and that reduced lipid levels at admission correlate with disease severity. No significant differences were found in triglyceride, HDL, and LDL levels, possibly reflecting the influence of age, diet, comorbidities, and interindividual variability [52, 53].

Increase in blood when glomerular filtration declines; eGFR is a key measure of renal filtration [58]. Urea and creatinine levels were higher in COVID‐19 patients, while eGFR and albumin were significantly lower. Elevated BUN at admission [e.g., > 16.05 mg/dL] has been associated with increased mortality risk [59]. Ok et al. [60] similarly found that higher BUN levels correlate with severe disease. Tian et al. [61] showed elevated BUN and creatinine in severe COVID‐19. The literature indicates a higher risk of acute kidney injury in COVID‐19 and its negative prognostic implications [62, 63]. Zheng et al. [64] found that elevated creatinine is associated with severe disease and higher in‐hospital mortality, and it has been suggested that such patients require closer ICU and mechanical ventilation surveillance [65]. In another study of 121 COVID‐19 positive and 53 negative individuals, higher CRP, older age, longer symptom duration, higher BUN and BUN/creatinine ratio, and lower eGFR were associated with renal impairment [66]. Lim et al. also reported decreased eGFR among a large SARS‐CoV‐2 cohort [67]. In line with these data, elevated urea and creatinine and reduced eGFR in this study suggest impaired renal function in the patient group. No difference in total protein was observed, consistent with findings by Turnic et al. [32]. No significant differences in uric acid levels were found, in agreement with observations that uric acid may fluctuate during ICU follow‐up without consistent early‐phase changes [68]. Tian et al. reported variable albumin findings, while Rabbani and Ahn [69] highlighted that hypoalbuminemia may downregulate ACE2 and exacerbate RAAS activation and lung injury. In the present study, albumin levels were decreased in the COVID‐19 group.

Electrolytes [Na, K, Cl, Ca, P, Mg] reflect fluid–electrolyte balance and neuromuscular stability. In this study, electrolyte values were within reference ranges. Santotoribio et al. [70] reported normal Na and K distributions across sex‐stratified groups. In contrast, Vásquez‐Procopio et al. [71] observed increased Mg and Ca and decreased Na in pregnant women with COVID‐19. Other studies have reported decreased Ca and hyponatremia in subsets of COVID‐19 patients [72, 73]. Taheri et al. [74] noted low Na values in a proportion of cases, and Na levels have been reported lower in COVID‐19 pneumonia than in non‐pneumonic cases [75]. In the present study, a low‐to‐moderate negative correlation between Apelin‐13 and Na was observed only in the patient group; no prior study specifically evaluating Apelin–Na interactions in COVID‐19 was identified.

Consistent with previous reports [76, 77], a statistically significant increase in glucose levels was observed in the patient group. Ardestani and Azizi proposed that SARS‐CoV‐2 triggers mitochondrial ROS and HIF‐1α activation, enhancing glycolysis and elevating intracellular glucose [78]. A meta‐analysis by Chen et al. [40] associated hyperglycemia with COVID‐19. In this study, a low‐to‐moderate positive correlation between Apelin‐13 and glucose was found in the COVID‐19 group. Kenoosh and Awad [76] reported higher Apelin and glucose levels in COVID‐19 patients and a positive correlation between them. Yue et al. [79] previously demonstrated that Apelin improves insulin sensitivity.

No significant changes in serum amylase and CK levels were observed between groups. Pribadi and Simadibrata [80] found elevated amylase mainly in severe COVID‐19. De Rosa et al. [81] and Friedman et al. [82] reported CK elevations in subsets of patients, associated with disease severity. In the present study, first‐sample measurements after diagnosis may partly explain the absence of marked differences.

Significantly increased CRP levels were observed in the patient group. Mosquera‐Sulbaran et al. [83] suggested that SARS‐CoV‐2‐related RAAS dysregulation increases Ang II and CRP, promoting inflammation and tissue damage. Poggiali et al. [84] also observed elevated CRP in COVID‐19. CRP is a classical acute‐phase reactant and key marker of systemic inflammation [85, 86]. Ferrari et al. [87] found higher CRP in COVID‐19 positives than negatives, while Shang et al. [45] reported higher CRP in severe disease. These findings, in line with our results, support CRP as an indicator of COVID‐19 severity. Gal‐3 is a lectin modulating inflammation and fibrosis [26], and Apelin‐13 is involved in cardiovascular and metabolic homeostasis [22, 29]. Although several studies have explored Gal‐3 and Apelin as prognostic markers in COVID‐19 [5, 27, 32, 88], our data indicate that Apelin‐13 and Gal‐3 did not show sufficient discriminative performance for COVID‐19 diagnosis in ROC analysis [low AUC values], and may not serve as standalone biomarkers.

## 5 Conclusion

As a result of the research, it is thought that Apelin‐13 and Gal‐3 alone cannot be determined as a distinctive parameter in the diagnosis and prognosis of COVID‐19 disease, but their relationship with routine biochemical parameters cannot be denied, and therefore they should be evaluated with different parameters in further studies.

## Funding

The author has nothing to report.

## Ethics Statement

This study was conducted following the approval of the Kafkas University Faculty of Medicine Non‐Interventional Clinical Research Ethics Committee (Decision Date: 08 June 2022, Session No.: 05, Decision No.: 13, Document No.: 80576354‐050‐99/49). The research was carried out in accordance with the principles of the Declaration of Helsinki and the Good Clinical Practice (GCP) Guidelines. Clinical and biochemical data collection was performed at the Chest Diseases Clinic and COVID‐19 Unit of Kars Harakani State Hospital, and biochemical analyses were conducted at the Central Biochemistry Laboratory of Kars Harakani State Hospital and the Department of Medical Biochemistry, Kafkas University Faculty of Medicine, under the necessary institutional permissions.

## Conflicts of Interest

The author declares no conflicts of interest.

## Data Availability

The data that support the findings of this study are available on request from the corresponding author. The data are not publicly available due to privacy or ethical restrictions.
